# Gardner Wells tongs modification in pre-operative management for cervical facet dislocation: A case report

**DOI:** 10.1016/j.amsu.2020.10.050

**Published:** 2020-10-29

**Authors:** S.Dohar AL. Tobing, Aryo Winartomo

**Affiliations:** a Orthopaedic Spine Consultant, Department of Orthopaedic & Traumatology, Cipto Mangunkusumo National Central Hospital and Faculty of Medicine, Universitas Indonesia, Jalan Diponegoro No. 71, Jakarta Pusat, Jakarta, 10430, Indonesia; b Resident, Department of Orthopaedic & Traumatology, Cipto Mangunkusumo National Central Hospital and Faculty of Medicine, Universitas Indonesia, Jalan Diponegoro No. 71, Jakarta Pusat, Jakarta, 10430, Indonesia

**Keywords:** Cervical facet dislocation, Cervical traction, Preoperative management, Case report

## Abstract

**Introduction:**

Cervical facet dislocations are one of the traumas that caused the neurological disability, and it is often found and shows a spectrum of facet fracture-dislocations. Cervical facet dislocation classified by the mean of mechanism into a flexion-distraction injury. The goal of the treatment is to reduce the dislocation in favour of the patient's condition and hospital facility.

**Method:**

We reported a case of 32 years old female with incomplete spinal cord injury due to Flexion distraction injury of C4–C5 spine, cervical X-Ray shows anterior translation for about 50% of C4 relative to underlying C5 on lateral projection, the patient was diagnosed with bilateral facet cervical dislocation and treated by gradual closed reduction using Gardner Wells Tongs followed by posterior body stabilization and fusion.

**Results:**

We initially load of 4 kg gradually along with continuous observation using lateral cervical radiograph and careful neurological assessment. The dislocation was finally reduced after gradual and dynamic loading with 14 kg load.

**Discussion:**

There are several strategies for managing cervical injuries. Aside from whether the MRI has to perform before or after the reduction, the option on whether to use closed or open reduction can be managed at best in favour of the current condition.

**Conclusion:**

Gardner Wells tongs is one of the best alternatives when the surgical approach is unavailable.

## Highlights

• The dislocation reduced using gradual and dynamic loading with 14 kg load.
• Gardner Wells tongs is one of the best alternatives to surgery.
• Gradual and dynamic loading proof to be safe and effective.

## 1 Introduction

Cervical facet dislocation has been reported in the adult population, and the injuries are caused mainly after fall and causing low energy ground levels or after high-trauma such as collision with motor vehicles or diving accidents. It accounts for 5% of all traumatic cases, and about one-third of the patients coincides with spinal cord injury, which makes these cases devastating in the manner of early and late complications [1]. Vaccaro and his associates are suggesting three variables to evaluate the stability of cervical injury: the fracture morphology, the integrity of the posterior ligament complex, and the neurological status [2].

The cervical facet dislocation mainly happens between two mechanisms: an excessive flexion-distraction or flexion-rotation, and both are causing one or both of the inferior facets of the superior vertebra to shift anteriorly to the superior facet of the vertebra below. Lower cervical facet dislocations usually coincide with disruption to the anterior or posterior element of the cervical vertebra soft tissue such as longitudinal ligaments, ligamentum flavum, annulus fibrosus, interspinous ligaments, and apophyseal joint ligaments. The two previously mentioned mechanisms arise potential for translation and dislocation because of the smaller diameter and lower height of the superior articular process combine with a more horizontally oriented inferior articular process [3].

Traditionally, the radiographic evaluation of suspected patients with cervical facet dislocation consists of a cross-table lateral view in addition to plain radiographic views. The extent of the soft tissue damage could be evaluated using MRI, although the whole division of the injury still divided into unilateral or bilateral dislocation. The unilateral facet dislocation is defined when the translation of the cephalad vertebrae by 25% of the anteroposterior diameter of the vertebral body on the imaging, whereas more than 50% translation is considered as bilateral facet dislocations. Some injuries are easily seen by using lateral radiograph alone, and some are hard to see, such as with AP views, oblique views, or open-mouth odontoid view [4].

The goal of the treatment in spine injury is to recover the neurological damage and to stabilize as to reconstruct the spinal segments. The most common choice to stabilize the cervical spine is by approaching a close reduction method, and when it fails, the open reduction is preferred. Acute realignment of cervical trauma such as using Gardner Wells Tong remains the mainstay procedure followed by anterior, posterior, or combined fusion, as the method has gained acceptance in recent years [5].

In this report, we presented a case of 32 years old female admitted to our emergency department with altered consciousness post motor vehicle accident with clinical tetraparesis suspected due to cervical injury 2 hour before admitted. We assessed patient with bilateral cervical facet dislocation and patient underwent gradual closed reduction using Gardner Wells Tongs traction. Informed consent has been given by the patient to be reported in a case report. This paper has been reported in line with the SCARE criteria [6].

### 1.1 Case illustration

We presented a case of 32 years old female referred to our Cipto Mangunkusumo Hospital (RSCM) Emergency Department with altered consciousness following by a motor vehicle accident. Two hours before the admission, she was walking across the road when a high-speed motor bike suddenly hit her form her right side. She fell and was unconscious. Then, she was brought immediately to PGC Cikini Hospital and diagnosed as Moderate Head Injury. She was resuscitated using fluid therapy, and they put her into sleep using a sedative. After initial resuscitation, her family asked to refer her to RSCM. When she arrived at the ED, we immediately put her a cervical neck collar and do a few examinations such as head and neck X-ray, FAST, and Head CT-scan. No bleeding or vomiting was observed.

**Fig. 1:**
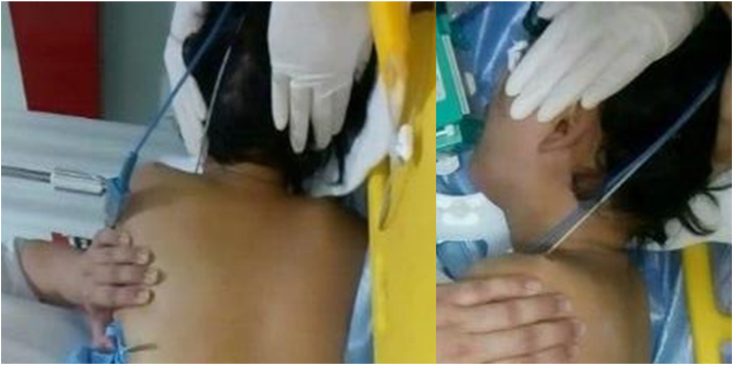
Physical examination of the neck.

The patient was presented with a mainly stable haemodynamic with altered mental status. Local examination of the neck shows no deformity, swelling, bruise, and all normal skin color (Fig. 1). On palpation, all pulsation on the radial artery was regular, yet despite unable check on tenderness, we felt a step off the sign. We avoid moving the head until we obtained the cervical radiograph. We immobilized the patient's neck to protect it by using a hard rigid cervical collar. The sensory and motor function on this patient cannot be assessed because of the previously used sedatives.

When the patient regained her consciousness, she felt numbness and tingling at her four extremities. She also felt weakness using both arms and legs, and she felt that her right half of the body is weaker than the other half. Her urination and defecation control were all normal. On the reflex examination, the patellar tendon and Achilles reflex all in normal function, yet there is a positive sign of Babinski reflex. Her laboratory results showed an increase of Leukocyte levels in 19.600 with possibility due to sterile inflammation, and the patient has Hyponatremia in 133 levels of Natrium. All of the other results are normal.

**Fig. 2:**
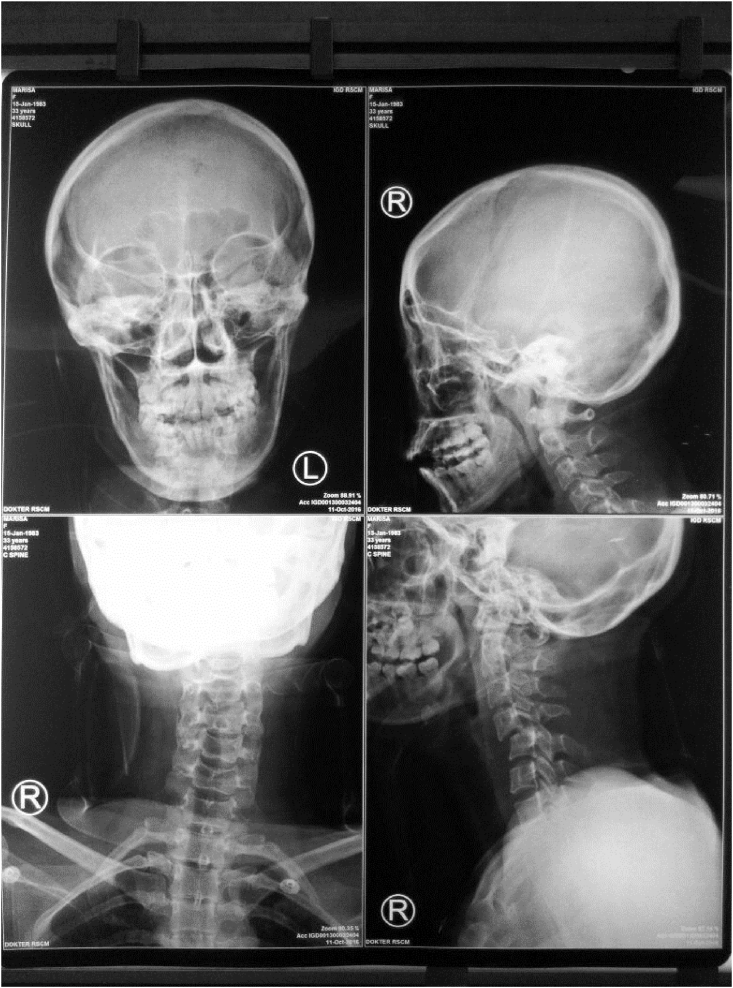
AP and Lateral radiograph of cervical spine.

The Cervical X-Ray showed less than 50% of anterior translation of C4 relative to underlying C5 on lateral projection (Fig. 2). From the Brain CT Scan, we observed that there was no focal haemorrhage, no skull fractures, yet there was an oedematous white matter that confirmed with diffuse axonal injury. The FAST or abdominal trauma ultrasonography was performed and showed no apparent abnormality.

The cervical CT-Scan was planned, and at first, we were planning to perform closed reduction with the possibility of an open reduction under general anesthesia. Still, the patient refused due to her troubling economic condition. We concluded that the patient has unilateral cervical facet dislocation, and we choose to perform gradual closed reduction using Gardner Wells Tongs.

**Fig. 3:**
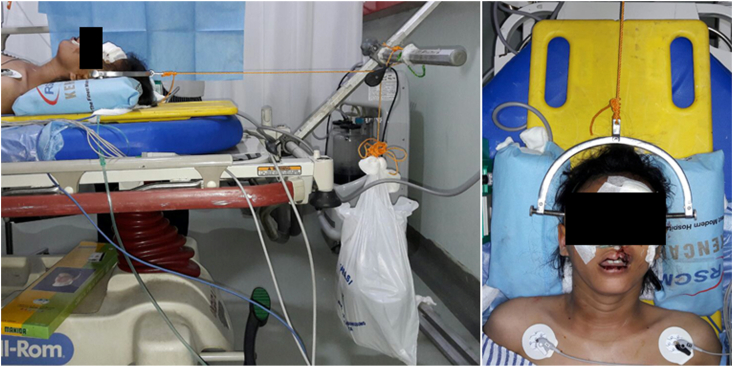
Garner Wells Tongs traction.

We applied an initial 4 kg load, followed by a careful haemodynamic and neurologic status assessment (Fig. 3). Right one hour after that, we obtained her a cervical radiograph to evaluate her improvement. The load was then added gradually, and with every added weight, we evaluated her haemodynamic and neurologic status along with lateral cervical radiograph serials.

**Fig. 4:**
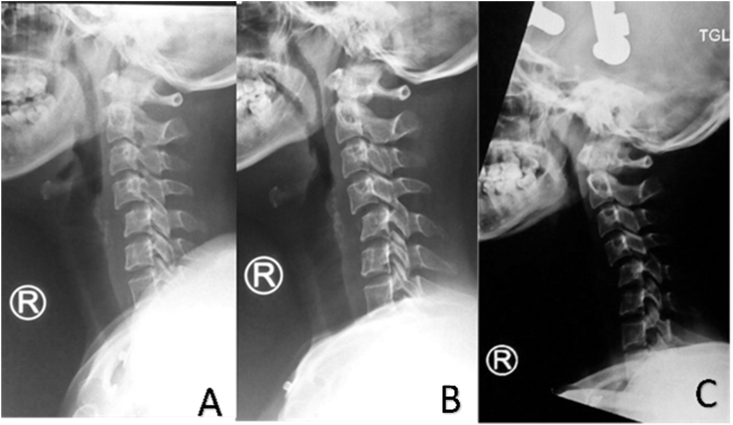
X-Ray of Spine on Gartner Wells Tongs.

**Fig. 5:**
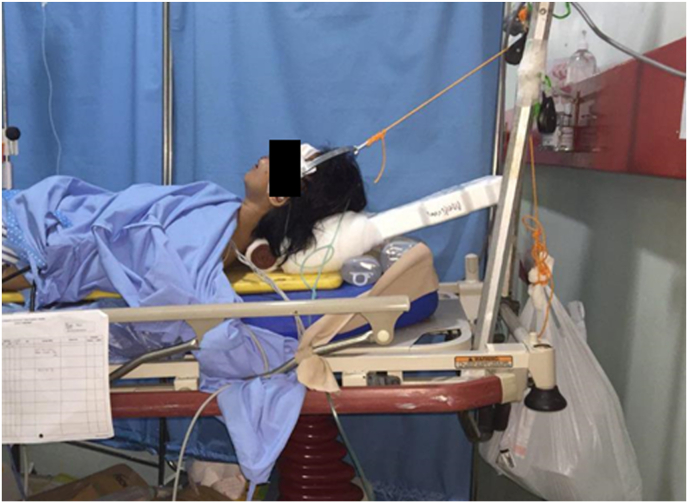
Reposition of traction vector.

After three times of additional load, we evaluated and concluded that there was no improvement in her cervical facet dislocation (Fig. 4). We changed her position right after to semi fowler, and the traction was repositioned in 30° relative to neck alignment to release facet locking (Fig. 5).

**Fig. 6:**
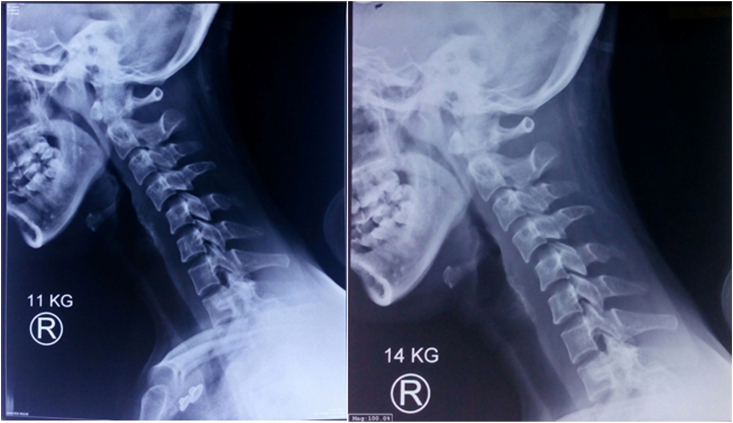
Lateral Radiograph of Cervical Spine.

After changing her position, her dislocation was finally reduced with 14 kg of load (Fig. 6). We turn back her position into a supine position and remove the amount to 5 kg. The traction was stand by still until five days and removed after no observed complication. The neck was still immobilized using a hard Philadelphia collar neck, and the patient was immediately planned for elective posterior stabilization.

**Fig. 7:**
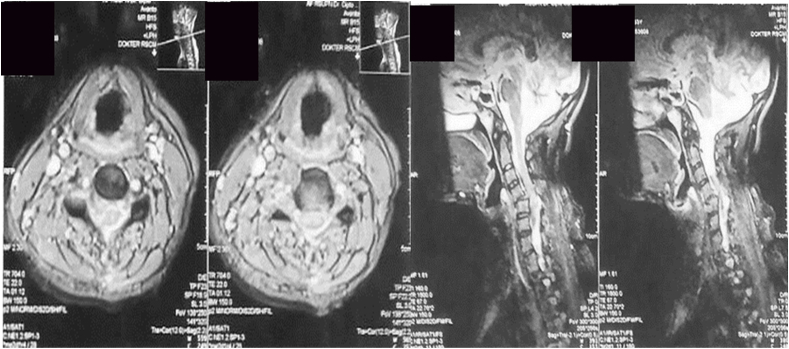
MRI of cervical spine.

One month later, the patient was agreed with the social department, and they were agreed on the cost of her operation. She was then performed an MRI of the cervical spine, and the results showed flexion distraction of C4–C5 with spondylolisthesis C4 grade 1, bilateral facet dislocation, and anterior and posterior ligamentous injury (Fig. 7).

**Fig. 8:**
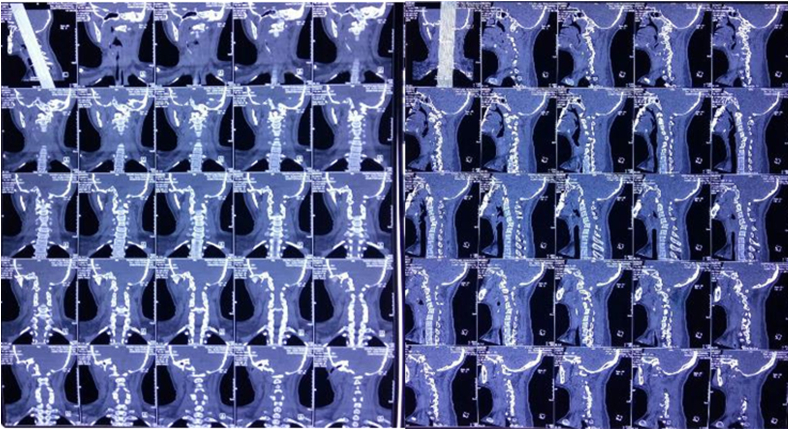
CT scan of cervical spine.

The CT scan of the cervical spine also showed spondylolisthesis of C4 to C5 towards the anterior side with compression of the spinal canal at the C5 level and subluxation of atlanto-axial C1–C2 (Fig. 8).

**Fig. 9:**
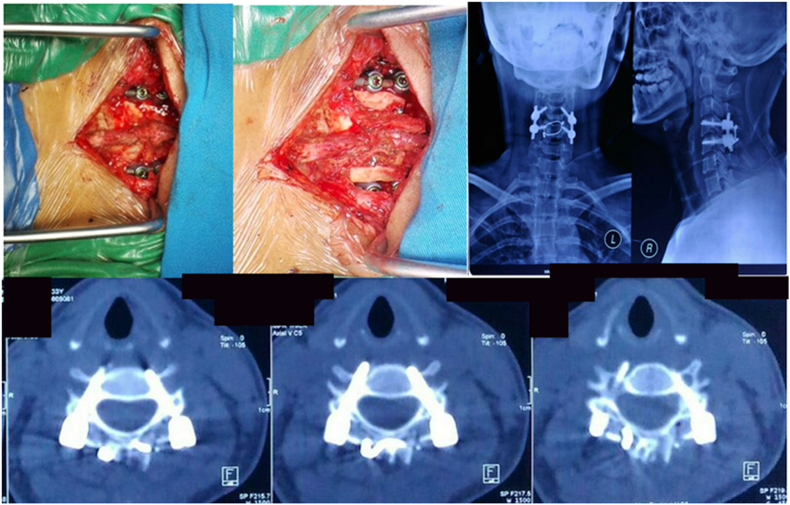
(A) Intra operative : screw placement (B) Intra operative : tricortical bone graft placement (C) Post operative : Anteroposterior and Lateral radiograph of cervical spine (D) Post operative : CT scan of cervical spine.

We decided to perform posterior stabilization and posterior fusion with tricortical bone graft. For posterior stabilization, we use four pedicle screw 3.5/20 and rod 40 mm two sides, and to achieve a posterior fusion, we place a tricortical bone graft from the right iliac wing (Fig. 9). Motoric and sensory status was continuously evaluated post-operatively, and there is a progression of the neurologic function of her lower extremity.

## 2 Discussion

The cervical spine fracture-dislocation remained a significant challenge in cases of trauma patients due to imminent risk of neurological complications and its related to the timing and modality of surgical management. There were no guidelines that have been agreed on the treatment of unilateral facet dislocation. However, several strategies for treatment have been proposed [7].

Recent studies showed possible herniation disc injury that occurs more than half on cases of unilateral facet dislocation and more than 80% of bilateral dislocations [8]. These herniated discs will pose a greater risk of neurological deterioration due to the compressed spinal cord that will result in spinal cord injury. One strategy to avoid this is to perform the MRI immediately after the patient was admitted to the ED [9]. The use of MRI not only helps in diagnosis but also in planning possible management options and forecast the prognostic of the disease. The closed reduction management has been well established, yet the controversies remain on when to use the MRI in case of facet dislocation. The patient will expose to a risk of delayed treatment, and it's put on weight on the spinal cord imminent risk of injury and disability. On the other hand, the facet dislocation is forceful enough to damage the surrounding soft tissue structures, and this has led to the belief that protrusion of the nucleus can go unnoticed as few surgeons decide the treatment based on the image of the MRI [10].

If there are any disc protrusion present, the anterior discectomy is recommended before the reduction, followed by with or without anteriorly interbody fusion and plate [11]. Another strategy as an alternative in an awake, alert, and cooperative patient is to use closed reduction with a cranial tong. It must be followed with MRI before the patient undergo surgical intervention to access any traumatic disc herniation or other disc occupying lesion [12].

Regardless of using the MRI before or after the reduction, from study to study, there is various management for cervical facet dislocations, and there is a 2-step process universally accepted to treat the injury. First, the facet displacement was reduced, and this was performed non-operatively using traction such as Halo ring or Gardner-Wells tongs, as many previously mentioned, or operatively via open reduction. And the second is dependent on the success of the closed reduction. The patient was either remain treated conservatively, immobilized the neck using a hard rigid collar neck, or underwent surgical fusion [13]. Kepler et al. found in their systematic review that operative treatment yields more success of anatomical reduction (90.8%) compared to a non-operative procedure (43.2%), regardless of their advantage and disadvantage, such as the risk of surgical procedure [14].

The decision on using closed or open reduction is based on the patient's condition, the facility, and resources of the institution and the physician's experience. Closed reduction only can be performed on a cooperative patient and followed by a serial neurological exam and continuous imaging every time the weight changes [15].

Following the surgical options, the anterior approach of open reduction involves the removal of the disc followed by fusion of the body vertebra. The advantages of this procedure are the ability to keep the patient supine and avoid repositioning in unstable cervical injury. The disc will be removed anteriorly, and decompression is performed. The disadvantage of this procedure is the inferior biomechanical control of instability post-operatively. Henriques et al. reported 7 out of 13 patients with bilateral facet dislocations treated with anterior approaches alone suffered postoperative recurrent subluxation [16]. Our patient was using the posterior approach, and it's the most common path to treating bilateral facet dislocations. The posterior approach has an advantage in stabilizing the unstable cervical segments, especially in flexion-distraction injuries with injury to the posterior longitudinal ligaments. The disadvantage is only flipping the patient and increase the risk of further trauma to the spinal cord. The posterior approach also restricts the visualization when decompressing the ventral epidural space [17].

Our patients were admitted to our Emergency Department with bilateral facet dislocation of C4-5 and were prepared to undergo surgery in an attempt to open reduction followed by posterior stabilization under general anesthesia. Yet the patient refused the option due to her financial problems. The patient was awake, and we offered a closed reduction using Gardner Wells tongs as an alternative. We add the load of 1 kg every half hour, and the reduction was achieved at 14 kg, and the patient continues stable haemodynamically, and neurological assessment was continuously evaluated in the process. After the reduction made, the load was reduced to 5 kg and maintained until five days to avoid recurrence, and no complication was observed. Contrary to this case report, Ye et al. reported failure to achieve reduction using Gardner Wells tongs in 36 patients with bilateral cervical facet dislocations [18]. The difference between our report, Ye et al. limit the maximum weight of traction to just only 10kg, while we increased the load every half our until the reduction was achieved. Other study from Visocchi et al. also reported failure of Gardner–Wells tongs. They use traction weight ranging from 3 to 5 kg depending on the age and the weight of the patient. They increase the weight up to one eight of the patient's weight, but none achieve reduction [19]. Although several studies reported failure of achieving reduction, a systematic review by Saleh et al. showed that Gardner Wells tongs were shown to be effective and safe to achieve reduction. Even in special population such as in transporting patient with aircraft or even in pregnant patient, little to no complication occurred [20].

The patient then undergoes elective posterior stabilization, and until that time, she was using the Philadelphia collar neck. All of her status, including hemodynamic and neurological, are continuously evaluated until the patient stable enough to undergo the operation procedure.

## 3 Conclusion

This is a case of bilateral facet dislocation of C4–C5 that achieved closed reduction by using Gardner Wells tongs traction. The gradual and dynamic loading proof that the procedure is safe, the reduction made, and become the best options when open reduction is unavailable due to various reasons.

## Ethical approval

This is a case report; therefore, it did not require ethical approval from ethics committee. However, we have got permission from the patients to publish his data.

## Sources of funding

No sponsorship for this case report.

## Author contribution

Singkat Dohar Lumban Tobing contributes in the study concept or design, data collection, analysis and interpretation, oversight and leadership responsibility for the research activity planning and execution, including mentorship external to the core team.

Aryo Winartomo contributes in the study concept or design, data collection, analysis and interpretation, writing the paper.

## Registration of research studies

This is not a first in man case report. Registration of research is not needed.

## Guarantor

Singkat Dohar Lumban Tobing is the sole guarantor of this submitted article.

## Consent

Written informed consent was obtained from the patient for publication of this case report and accompanying images. A copy of the written consent is available for review by the Editor-in-Chief of this journal on request.

## Funding sources

The authors report no external source of funding during the writing of this article.

## Informed consent

Written informed consent was obtained from the patient for publication of this case report and accompanying images.

## Provenance and peer review

Not commissioned, externally peer-reviewed.

## Declaration of competing interest

The authors declare that there is no conflict of interest regarding publication of this paper.

## Appendix A Supplementary data

Application Application
